# Quick Leukocyte Nucleus Segmentation in Leukocyte Counting

**DOI:** 10.1155/2019/3072498

**Published:** 2019-06-11

**Authors:** Yapin Wang, Yiping Cao

**Affiliations:** College of Electronic Information Engineering, Sichuan University, Chengdu, Sichuan 610064, China

## Abstract

The leukocyte nucleus quick segmentation is one of the key techniques in leukocyte real-time online scanning of human blood smear. We propose a quick leukocyte nucleus segmentation method based on the component difference in RGB color space. By analyzing the captured microscopic images of the peripheral blood smears from the autoscanning microscope, it is found that the difference values between B component and G component (B − G values) in the regions of the leukocyte nuclei and the platelets are much bigger than those in the other regions, even in the regions including the stains. So, the B − G values can segment the leukocyte nuclei and the platelets with an appropriate empirical threshold because the platelets are much smaller than the leukocyte nuclei, so the leukocyte nuclei can be segmented by size filtering. Also, only an 8 bit subtraction operation is performed for the B − G values, and it can improve the leukocyte nucleus segmentation speed significantly. Experimental results show that the proposed method performs well for the five types of leukocyte segmentation with a quick speed. It is very suitable for the real-time peripheral blood smear autoscanning test application. In addition, the five types of leukocytes can be counted accurately.

## 1. Introduction

The leukocyte is a kind of blood cell which can immunize humans from diseases, resist the invader, swallow foreign bodies, and produce antibodies. Thus, it has an inseparable relationship with human health [1–3]. So, leukocyte parameters are very important for human health diagnosis in clinical medicine. Presently, manual inspection is still regarded as the main leukocyte detection method in the most hospitals [4, 5]. With the development of computer science, image processing, and pattern recognition in recent years, research on leukocytes automatic analysis system made a great progress, which has gotten a good effect on actual medical application. Leukocyte nucleus segmentation is the most basic link in leukocyte automatic analysis system. The whole process is real-time collection and analysis. That is to say the image collection and image processing are almost at the same time. Therefore, for the leukocyte automatic analysis system, the faster the leukocyte nucleus segmentation speed is, the shorter the time consumption of the image processing will be.

In order to solve this problem, a number of image segmentation methods have been proposed, for example, Cao et al. proposed a leukocyte segmentation scheme interval-valued fuzzy sets [6], Hung et al. proposed a segmentation method based on leukocyte nucleus enhancer [7], Marzuki et al. proposed a leukocyte nucleus segmentation method by using active contour [8], Pan et al. proposed a leukocyte segmentation method by using simulated visual attention (SVA) [9], Sezgin and Sankur proposed the threshold segmentation which is used to segment leukocyte nucleus based on the S component image [10], Yang et al. proposed a method of leukocyte segmentation based on S component and B component image (SAB) [11], and Otsu algorithm is used in the S component to segment the leukocyte nucleus [12]. The threshold is only fit for the histogram that has a clear peak or multipeaks. When processing the image with multiobject or complex background, the threshold segmentation accuracy is sometimes not as expected. Ko et al. proposed a segmentation method combining different features and mean shift clustering algorithm based on the saliency map to segment nuclei; however, the cytoplasm boundaries are too weak to be precisely obtained [13]. Xing et al. proposed a learning-based framework for robust and automatic nucleus segmentation with shape preservation [14]. Madhloom et al. proposed a segmentation method by using morphological operations to extract the leukocytes from other cells and background [15]. Even though some segmentation methods perform well in accuracy, the majority of these methods have some defects to some extent, such as the rigorous condition of illumination, the complicated operation of arithmetic, the difficult confirmation of parameters, the long consumption of time, and so on. In this study, we focus on developing a quick leukocyte nucleus segmentation method in leukocyte counting which can satisfy the real-time peripheral blood smear autoscanning test. Firstly, subtraction operation is conducted between B component and G component to obtain grayscale image. It can save a large amount of computing time so as to improve the processing speed and minimize the stain disturbance in the leukocyte counting; then, the leukocyte nuclei are segmented with an empirical threshold set by lots of experimental analysis statistics; finally, the leukocyte nuclei are located accurately by using horizontal and vertical projection [16, 17].

## 2. Leukocyte Nucleus Segmentation

When using above existing segmentation methods for leukocyte nucleus segmentation, the segmentation results might be sensitively influenced by the surrounding conditions and dyeing conditions. Some methods may play higher segmentation accuracy but their segmentation speed may not be so fast to be used in real-time online leukocyte testing. Some methods play a convenient segmentation speed, but their segmentation accuracy may not be good for leukocyte nucleus segmentation. It is found that the leukocyte nuclei and platelets can be segmented by the B − G values of the captured blood smear microscopic images; because the platelets are much smaller than the leukocyte nuclei, the leukocyte nuclei can be segmented by size filtering. The final segmentation can perform accurately and quickly.

### 2.1. Segmentation Parameter Selection

**Figure 1:**
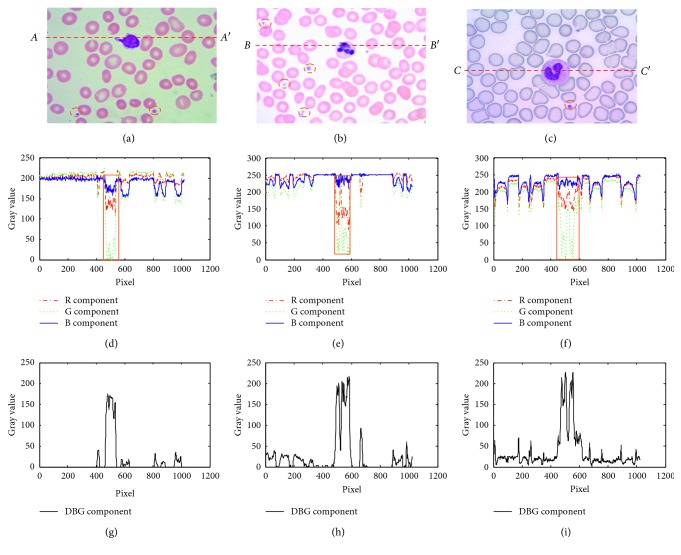
The R, G, and B components' cutaway views for captured leukocyte images at different conditions. (a) The captured leukocyte image 1. (b) The captured leukocyte image 2. (c) The captured leukocyte image 3. (d) The R, G, and B components' cutaway views on *AA*′ of (a). (e) The R, G, and B components' cutaway views on *BB*′ of (b). (f) The R, G, and B components' cutaway views on *CC*′ of (c). (g) The B − G component's cutaway view on *AA*′ of (a). (h) The B − G component's cutaway view on *BB*′ of (b). (i) The B − G component's cutaway view on *CC*′ of (c).

Figures 1(a)–1(c) show three typical captured leukocytes at different surrounding conditions and different dyeing conditions.  Figure 1(d) shows the R, G, and B components' cutaway views on the dashed line *AA*′ in Figure 1(a).  Figure 1(e) shows the R, G, and B components' cutaway views on the dashed line *BB*′ in Figure 1(b).  Figure 1(f) shows the R, G, and B components' cutaway views on the dashed line *CC′* in Figure 1(c). The bold solid line rectangle ranges in Figures 1(d)–1(f) indicate the leukocyte nucleus' regions.  Figure 1(g) shows the B − G component's cutaway view on the dashed line *AA*′ in Figure 1(a).  Figure 1(h) shows the B − G component's cutaway view on the dashed line *BB*′ in Figure 1(b).  Figure 1(i) shows the B − G component's cutaway view on the dashed line *CC*′ in Figure 1(c). From Figures 1(d)–1(i), we can see that the B − G values in the regions of the leukocyte nuclei are always much bigger than those in other regions whether the surrounding conditions and dyeing conditions change or not. So, we can use B − G values to segment the leukocyte nuclei. From Figures 1(a)–1(c), we can also see that the dashed circled platelets have almost the same color as the leukocyte nuclei in the same images whether the surrounding conditions and dyeing conditions change or not, so the B − G values can segment both the leukocyte nuclei and platelets at the same time; because the platelets are much smaller than the leukocyte nuclei, the leukocyte nuclei can be segmented by size filtering. It means that B − G values can be selected as the parameters to segment the leukocyte nuclei robustly for different surrounding conditions and different dyeing conditions.

**Figure 2:**
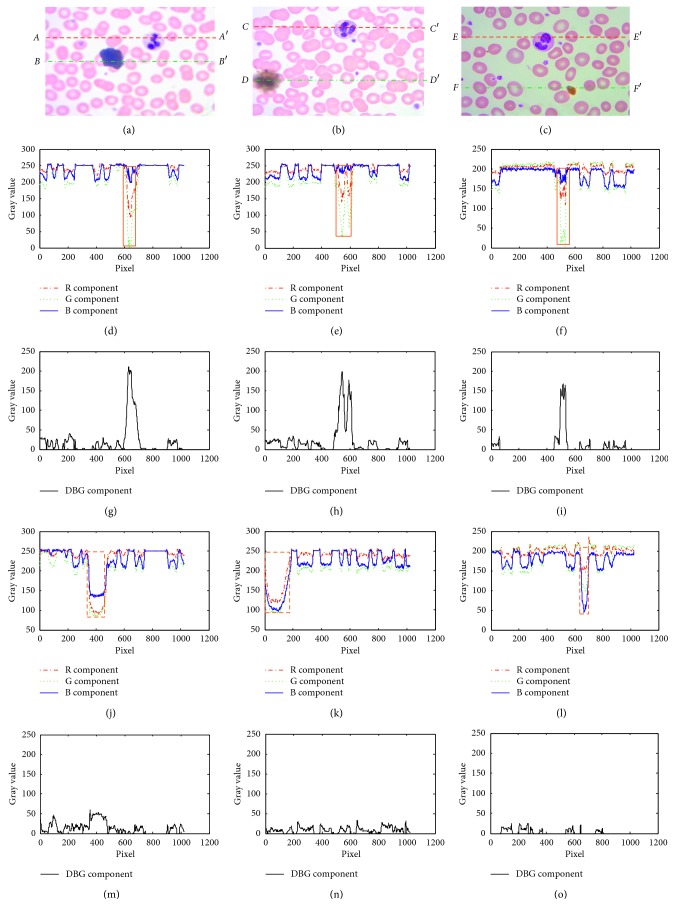
The R, G, and B components' cutaway views for captured leukocyte images with stains at different conditions. (a) The leukocyte captured image 1. (b) The leukocyte captured image 2. (c) The leukocyte captured image 3. (d) The R, G, and B components' cutaway views on *AA*′ of (a). (e) The R, G, and B components' cutaway views on *CC*′ of (b). (f) The R, G, and B components' cutaway views on *EE*′ of (c). (g) The B − G component's cutaway view on *AA*′ of (a). (h) The B − G component's cutaway view on *CC*′ of (b). (i) The B − G component's cutaway view on *EE*′ of (c). (j) The R, G, and B components' cutaway views on *BB*′ of (a). (k) The R, G, and B components' cutaway views on *DD*′ of (b). (l) The R, G, and B components' cutaway views on *FF*′ of (c). (m) The B − G component's cutaway view on *BB*′ of (a). (n) The B − G component's cutaway view on *DD*′ of (b). (o) The B − G component's cutaway view on *FF*′ of (c).

But actually, when preparing the blood smear, the stains are unavoidable. Figures 2(a)–2(c) show three typical captured leukocytes with stains at different surrounding conditions and different dyeing conditions. Figures 2(d)–2(f), show the R, G, and B components' cutaway views on the dashed lines in Figures 2(a)–2(c), respectively, which include the leukocyte nucleus regions (bold solid line rectangles). Figures 2(g)–2(i) show the B − G component's cutaway views on the dashed lines in Figures 2(a)–2(c), respectively, which include the leukocyte nucleus regions. Figures 2(j)–2(l) show the R, G, and B components' cutaway views on the dashed dotted lines in Figures 2(a)–2(c), respectively, which include the stain regions (bold dashed line rectangles). Figures 2(m)–2(o) show the B − G component's cutaway views on the dashed dotted lines in Figures 2(a)–2(c), respectively, which include the stain regions. By comparing them, the B − G values in the stain regions are alterable in a certain numerical range which are smaller than 50 concluded by lots of experimental analysis statistics while those in the regions of the leukocyte nuclei are always bigger than 110, so we can set an appropriate empirical segmentation threshold *T*_0_ (*T*_0_ ∈ [50, 100]) to minimize the stain disturbance at different surrounding conditions and different dyeing conditions. So, the B − G values used as the segmentation parameters show their good stability and robustness.

### 2.2. Algorithm Analysis for the Leukocyte Nucleus Segmentation Method

**Figure 3:**
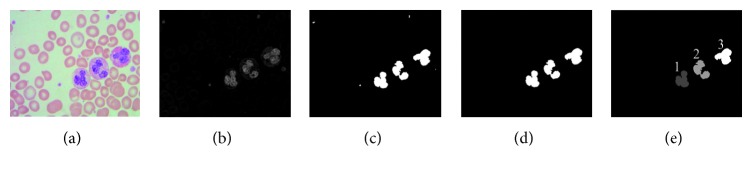
The connected region labeling image. (a) The multileukocyte captured image. (b) The B-G image. (c) The binarization image. (d) The segmented nucleus. (e) The connected region labeled image of (d).

Because only an 8 bit subtraction operation is performed for the B − G values, it has the shortest time consumption in all existing operations such as initialization operation, float operation, and double operation. Also, the segmentation threshold need not be calculated by some algorithms such as Otsu's algorithm and mean shift clustering algorithm; it is set to be *T*_0_ by experience through the experiments of thousands samples of leukocyte nuclei images. This method can avoid the long time consumption of searching for the segmentation threshold. Although the size filtering must be done, it is a common shared operation for all leukocyte segmentation methods. So, the total consumption time is still the shortest as well which means that the proposed method is a quick leukocyte nucleus segmentation method recently. The basic process of the leukocyte nucleus segmentation algorithm is as follows:

(1) Extracting the B component and G component, respectively.
(2) Making the subtraction operation between the B component and G component to obtain the gray image.
(3) Getting the binarization image with an appropriate empirical threshold for the gray image.
(4) Eliminating the platelets by size filtering to get the segmentation result.
(5) Labeling the connected regions with different gray values for the segmentation result as shown in Figure 3; after leukocyte nucleus segmentation, if there are more than one segmented leukocyte nucleus regions in an image, the judgment of lobular nuclei will be made. Firstly, the areas of all the leukocyte nucleus regions will be calculated. The maximal statistic area value of lobular region is *S*_0_. The segmented region whose area is larger than *S*_0_ will not be a lobocyte. For the rest regions, the empirical value of the Euclidean distance between the centroids is adopted to judge the lobular nuclei. The Euclidean distance *D* between the centroids of any two regions is calculated. If the distance *D* is bigger than the distance empirical value *D*_0_ (set by large numbers of experimental statistical), it means that the two regions are two independent leukocyte nuclei. Otherwise, they belong to the same leukocyte nucleus. The above steps are repeated until all the regions have been judged.
(6) Making the projection operation for the each labeled connected region to obtain the envelope by using the following equations:
  (1) $$\begin{array}{c} \left(x_{\text{l}},x_{\text{r}}\right)=\text{Prj}\left\{I_{\text{BGS}}\right\}, \end{array}$$
  (2) $$\begin{array}{c} \left(y_{\text{t}},y_{\text{b}}\right)={\text{Prj}}_{x}\left\{I_{\text{BGS}}\right\}, \end{array}$$
  (3) $$\begin{array}{c} \left\{\begin{array}{c} x_{\text{c}}=\text{INT}\left\{\frac{x_{\text{l}}+x_{\text{r}}}{2}\right\},\\ y_{\text{c}}=\text{INT}\left\{\frac{y_{\text{t}}+y_{\text{b}}}{2}\right\}, \end{array}\right. \end{array}$$
  (4) $$\begin{array}{c} w=x_{\text{r}}-x_{\text{l}}, \end{array}$$
  (5) $$\begin{array}{c} h=y_{\text{t}}-y_{\text{b}}, \end{array}$$

where the *x*_l_, *x*_r_, *y*_t_, and *y*_b_ are the leftmost boundary, rightmost boundary, topmost boundary, and bottommost boundary, respectively, *I*_BGS_ is the segmented nucleus, Prj_*y*_ {.} and Prj_*x*_{.} are the vertical projection operator and the horizontal projection operator, respectively, (*x*_c_, *y*_c_) is the center of each leukocyte nucleus, the operator INT{.} is the meaning of integralization operator, and *w* and *h* are the width and the height of each leukocyte nucleus, respectively.

**Figure 4:**
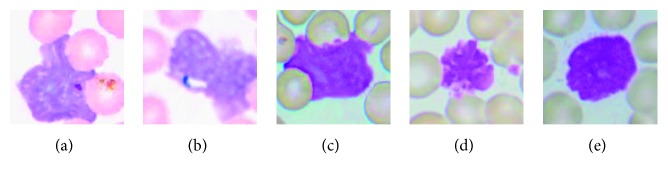
Some of the distinguished artifacts. (a) Artifact 1. (b) Artifact 2. (c) Artifact 3. (d) Artifact 4. (e) Artifact 5.

In case of important artifacts which are characterized by same color and size with the leukocyte nucleus, a judgment based on leukocyte feature is made to distinguish a leukocyte from an artifact after the real-time obtaining of the segmented images [18, 19]. The ratio of areas of cytoplasm and nucleus is the selected leukocyte feature. According to lots of experimental analysis statistics, the minimum value of the ratio of areas of cytoplasm and nucleus is 0.26 for leukocyte. If the ratio of areas of cytoplasm and nucleus is smaller than 0.26, the segmented image will be regarded as an artifact and be discarded. In order to obtain the region of cytoplasm, Yang's method using B component and saturation (S) component is adopted [11].  Figure 4 shows some of the segmented artifacts, and they are distinguished by the above mentioned method.

**Figure 5:**
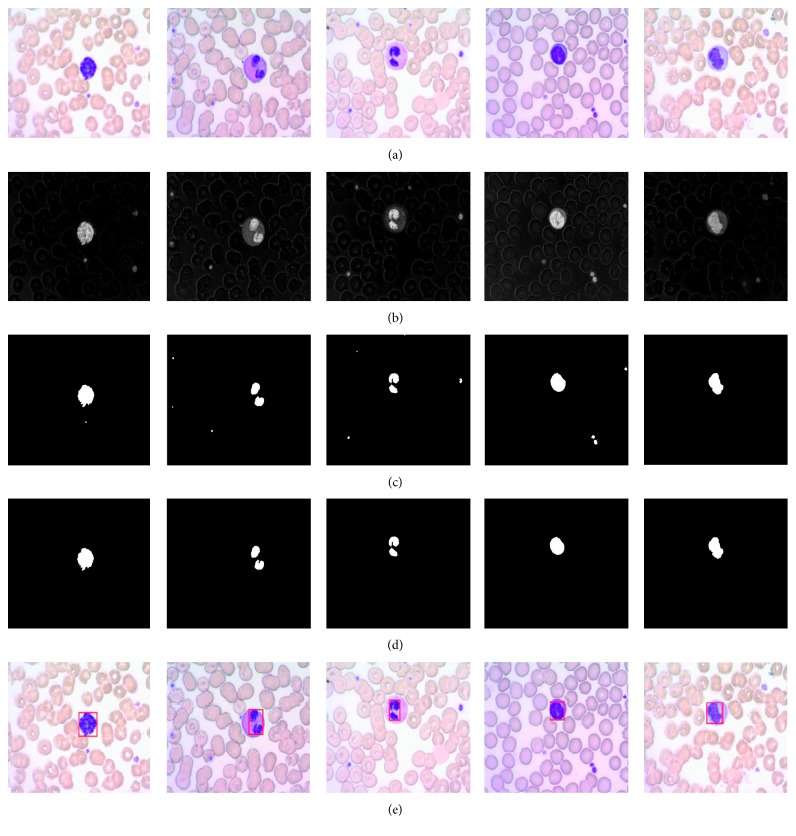
The segmentation process. (a) The captured leukocyte images. (b) The B-G images. (c) The binarization images. (d) The segmentation results. (e) The leukocyte nucleus location.

Thousands of leukocyte micrographic images are selected as experimental samples. Five typical different leukocytes from those samples are shown in Figure 5(a). They are described as in Rezatofighi and Soltanian-Zadeh [20]; Chan et al. [21]; and Rezatofighi and Soltanian-Zadeh [20]. They are basophil (Bas), eosinophil (Eos), neutrophil (Neu), lymphocyte (Lym), and monocyte (Mon) from left to right, respectively. The Bas has large granules which are dyed deep blue to purple and are often so numerous to mask the nucleus; the Eos has large granules which are acidophilic and appear pink (or red) after dyeing; the Neu has very tiny dyed granules with low visibility, and its nucleus is frequently multilobed with lobes connected by thin strands of nuclear material; the Lym has a very clear cytoplasm that is pale blue when dyed; the Mon is the largest among the leukocytes, and its nucleus is often “U” shaped or kidney bean shaped while its cytoplasm is abundant and light blue. Figures 5(b)–5(e) show the intermediate results processed by the proposed method. We can find that the nuclei for the five types of leukocyte can be located ideally though the surrounding conditions or the dyeing conditions are different. It shows the robustness of the proposed leukocyte nucleus segmentation algorithm. If an auxiliary analysis is performed with some other characteristics of the leukocyte, the five types of leukocytes can be classified and counted effectively.

## 3. Experimental Result Analysis

**Figure 6:**
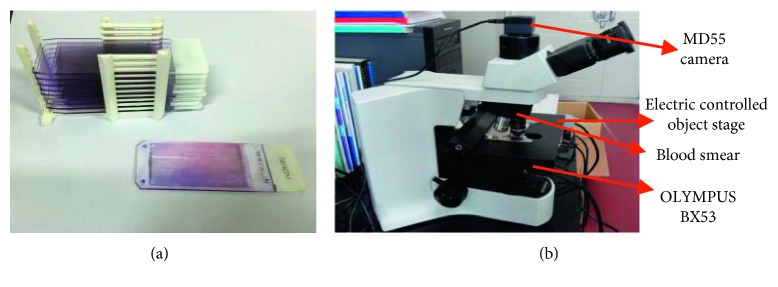
The blood smears and the autoscanning system experimental device. (a) Blood smears. (b) Autoscanning system experimental device.

All the algorithms were implemented with Visual C++ 6.0 on a Windows XP operating system with a 3.30 GHz Intel Core i3-3220 CPU and 4 GB memory. Peripheral blood smears are prepared with Wright-Giemsa dye shown in Figure 6(a). More than 3000 different microscopic images with 1024 ∗ 768 sizes were captured by a CCD camera, and they were collected from the People's Hospital of Sichuan Province. The experimental system is shown in Figure 6(b), an improved OLYMPUS BX53 microscope which can realize autocapturing leukocyte image with a MD55 camera, autofocusing by electrical driving the stage up and down and autoscanning by electrical driving the stage rightward and leftward or forward and backward with a three-axis step motor controlling system.

In order to analyze the segmentation accuracy and the real-time performance of the proposed segmentation method, segmentation accuracy data and time consumption data are recorded and compared with the existing method SAB based on Otsu's algorithm [11] and Ko et al.'s method based on mean shift clustering [13]. The segmentation standard of the leukocyte nucleus is manually extracted by the specialist in hematology.

### 3.1. Leukocyte Nucleus Segmentation Experiment Results

The leukocyte nucleus segmentation accuracy of the three methods is shown in Table 1. Over 577 representative samples at different degrees of dyeing or different illumination conditions are chosen for analysis.

It is easy to find from the experimental results that all three methods perform well. When using the proposed method, the nucleus segmentation accuracies of the five types of leukocytes are all more than 90%. In spite of the small sample, the segmentation accuracies of basophils can also reach 88.2% using the SAB and Ko's method. The proposed method performs a little worse for Eos than the SAB, but the difference is very small. And the average segmentation accuracy of the proposed method is 97.40%, which is higher than that of the SAB (95.40%) and that of Ko's method (92.44%). The experimental results show the stability and high accuracy of the proposed method.

### 3.2. Leukocyte Nucleus Segmentation Speed Analysis

The average time consumptions for the five types of leukocytes by the three nucleus segmentation methods for the above 577 samples are shown in Table 2.

We can see from Table 2 that the average time consumption for the leukocyte nucleus segmentation by the proposed method, Ko's method, and the SAB method is 0.26 ms, 2.6 ms, and 27.15 ms, respectively. It reveals that the proposed method is ten times quicker than Ko's method and over one hundred times quicker than the SAB method. So, the proposed method has better timeliness to meet the real-time requirement in the blood smear autoscanning test application.

### 3.3. Leukocyte Autoscanning and Autocounting

**Figure 7:**
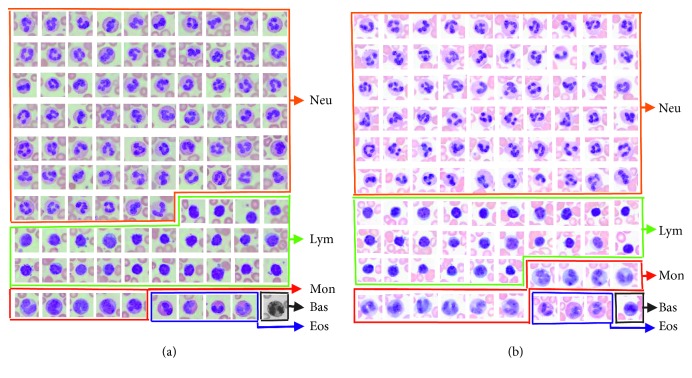
The leukocyte autoscanning test results for different blood smears at different conditions. (a) The extraction leukocyte images of blood smear sample 1. (b) The extraction leukocyte images of blood smear sample 2.

The quick segmentation technique has been applied in the real-time autoscanning for the leukocyte peripheral blood smear. Thousands of blood smear samples have been scanned automatically. In order to provide sufficient information for doctor to diagnose disease efficiently, 100 leukocyte images from each blood smear must be extracted by a real-time leukocyte autoscanning system.  Figure 7 shows the leukocyte autoscanning results for two of the blood smear samples at different surrounding conditions and different dyeing conditions. The leukocyte images are realistically well extracted.  Table 3 shows the leukocyte counting results for ten tested blood smear samples in which sample 1 and sample 2 are the same samples shown in Figure 7. We can see from Figure 7 that the proportions of Neu, Lym, Mon, Eos, and Bas are 66%, 24%, 5%, 4%, and 1%, respectively, in sample 1 while those in sample 2 are 60%, 26%, 10%, 3%, and 1%, respectively. In this way, the corresponding proportions of different samples can be concluded accurately. Also, the average time consumption for leukocyte counting is 100 (±1) seconds per blood smear; most of the time is spent for the mechanical movement while scanning. The segmentation time consumption in the real-time leukocyte autoscanning system might be less than 2 milliseconds per extracted leukocyte image, and thus it means that the proposed method can well meet the real-time application.

The experimental results show that the proposed segmentation method has good segmentation quality and quick speed; it has better stability and robustness.

### 3.4. Segmentation Results on Clumped Leukocytes

**Figure 8:**
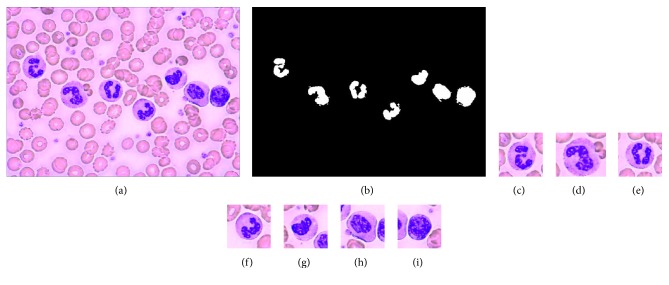
Clumped leukocytes and the segmentation result. (a) Clumped leukocytes. (b) Segmentation result. (c) Segmented image 1. (d) Segmented image 2. (e) Segmented image 3. (f) Segmented image 4. (g) Segmented image 5. (h) Segmented image 6. (i) Segmented image 7.

In order to verify the practicability of the proposed method on clumped human peripheral blood leukocytes, an experiment is conducted. As can be seen, the leukocytes are quite clumped in Figure 8(a). The segmentation result using our method is shown in Figure 8(b). The segmented images are Figures 8(c)–8(i). As can be seen, all the leukocyte nuclei can be segmented successfully. It indicates the practicability of the proposed method on clumped leukocytes. For a peripheral blood smear that meets the requirements, the overlap between leukocytes will not appear. We think that the proposed method will work well when there is no overlap between leukocytes in the image to be segmented.

## 4. Conclusions

A quick leukocyte nucleus segmentation method based on the B − G values in leukocyte counting has been proposed. Because only an 8 bit subtraction operation is performed for B − G values, it can reduce the amount of computation significantly. The average time consumption of the proposed method is only 0.26 ms. Experimental results show its feasibility, robustness, and the better real-time performance. The proposed method can be applied successfully in the real-time autoscanning test for the leukocyte peripheral blood smear which includes autoscanning, autofocusing, autoextracting, and autocounting of leukocyte images to reduce the errors caused by manual operation. So, it guarantees sufficient results for doctor to diagnose the diseases more quickly and more effectively.

## Figures and Tables

**Table 1:** Segmentation accuracy of the five types of leukocytes.

Types of leukocyte	Numbers of samples	Segmentation method					
		SAB (based on Otsu's algorithm)		Ko's method (based on mean shift clustering)		The proposed method	
		Result of segment	Accuracy (%)	Result of segment	Accuracy (%)	Result of segment	Accuracy (%)
Eos	45	44	97.8	41	91.1	43	95.5
Bas	17	15	88.2	15	88.2	16	94.1
Neu	246	242	98.4	232	94.4	245	99.6
Mon	138	133	96.4	129	93.8	136	98.6
Lym	131	126	96.2	124	94.7	130	99.2

**Table 2:** Time consumptions for the leukocyte nucleus segmentation.

Types of leukocyte	Numbers of samples	Average time consumptions for each leukocyte (ms)		
		SAB (based on Otsu's algorithm)	Ko's method (based on mean shift clustering)	The proposed method
Eos	45	27.83	2.42	0.26
Bas	17	26.72	2.00	0.27
Neu	246	27.59	3.51	0.24
Mon	138	27.14	3.17	0.25
Lym	131	26.45	1.91	0.26
Average		27.15	2.60	0.26

**Table 3:** The leukocyte counting results for ten tested samples (%).

Type	Sample									
	1	2	3	4	5	6	7	8	9	10
Neu	66	60	62	65	55	60	50	66	52	42
Lym	24	26	24	25	35	30	35	25	35	26
Mon	5	10	8	7	8	7	6	6	8	20
Eos	4	3	5	3	2	2	8	3	4	8
Bas	1	1	1	0	0	1	1	0	1	4

## Data Availability

The image data used to support the findings of this study are available from the corresponding author upon request.
